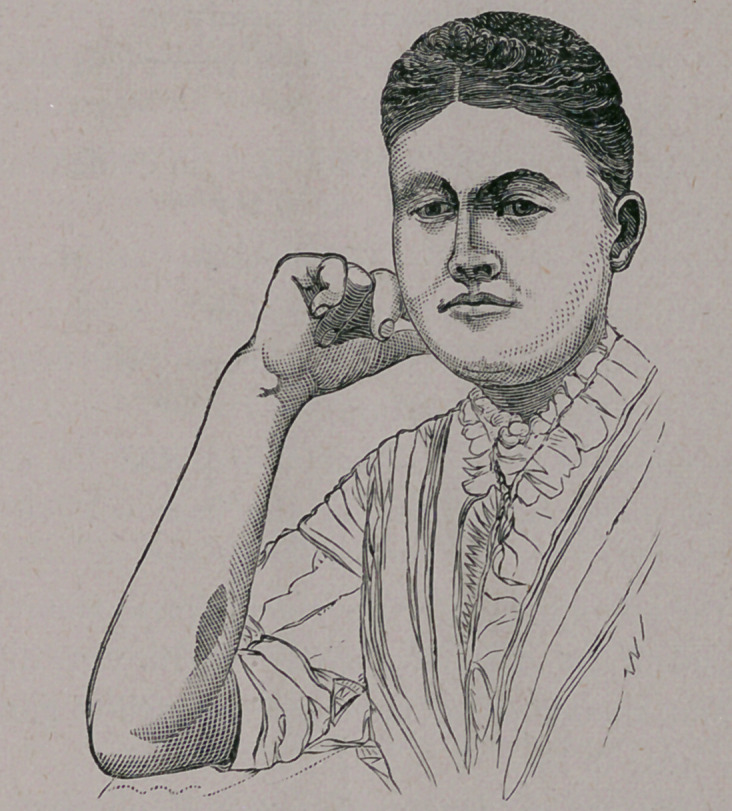# The Treatment of Ectropion by Transplantation of Skin*Reprint from the Transaction of the American Ophthalmological Society.

**Published:** 1881-04

**Authors:** Lucien Howe


### THE TREATMENT OF ECTROPION BY TRANSPLANTATION OF SKIN.*

BY DR. LUCIEN HOWE,

ALTHOUGH this method of operation is not entirely new to the profession, it has not, I believe, been formally brought to the notice of this society; and that fact, together with the apparent importance of the subject, would seem to warrant a few practical considerations concerning its application in a special instance, and the deductions to be made concerning it.

The ancient Indian plastic operation was simply modified by Tagliacozzi, when he took the flap from the arm instead of from the forehead or cheek, as had been previously done. In both instances, of course, an attempt was made to keep up the circulation till after the part had attached itself. But when M. Reverdin proved that small particles of skin, after being entirely removed, would again grow on an ulcerating surface, it was the demonstration of a new principle. This method has been known from the first to English readers as that of "grafting," and I think we should retain the term "transplantation," to indicate the transfer of large flaps having no pedicle to fresh wounds of corresponding size. This distinction is to a certain extent arbitrary, and one merges into the other, just as in practice the latter method was gradually developed from the former. They are both, however, of quite recent date, and so few names, comparatively, have thus far been identified with the different stages of the process, that it is not difficult to trace the transition..

Mr. Lawson† was probably the first to employ grafting for the treatment of ectropion, and even in his earliest cases, ten years ago, made use of particles as large as a three-penny piece, although Reverdin had recommended that they be much smaller. In 1872 Prof. Ollier‡ transplanted pieces still larger; the same

* Reprint from the Transaction of the American Ophthalmological Society.

† Lancet, Nov. 19, 1870.

‡ Bull. de l'acad., 2e Ser., I., 7, 1872.

year, Wecker's suggestion in the "Annales d'oculistique" indicated a further improvment; and in 1875 Dr. Wolfe reported two cases where the injured integument was replaced "by a larger flap dissected from the forearm, and entirely separated from its attachment." At the Fifth International Ophthalmological Congress, Dr. Wadsworth, of Boston, also demonstrated the advantages of this method. Two equally favorable cases have recently been reported by Dr. Aub, of Cincinnati, and one by Dr. Noyes, while Zehender has made two attempts in the same direction, with partial success. The transplantation of large flaps has therefore been confined to ophthalmic practice, and indeed the possibilty of such an operation seems to have been questioned or entirely ignored by some of the best surgeons.

Tne following passage from Holmes' Surgery illustrates the manner in which such attempts are usually regarded. The writer says: "Burger relates a case of partial success in the formation of a new nose on a lady, by a piece of integument completely cut away from the thigh; and Hoffacher, who was officially appointed to attend at the duels frequent among the students at Heidelberg, mentions some remarkable instances, which are attested by Chelius and Velpeau, of the reunion of parts completely sliced off by sword-cuts, *e. g.*, portions of the nose, lips, or chin. But no such license can be allowed in plastic surgery. The flap must retain its connection to the adjacent living structure by a pedicle, which is to be severed only after complete union and cicatrization of the raw surfaces."

The error of such statements has been sufficiently demonstrated by the results of cases already referred to, and I venture to add still another to the list, for the reason: 1, that, from all I can learn, the transplanted flap was larger than any thus far mentioned; 2, that the plan of procedure differed slightly from that adopted by others; and 3, that while the operation is still on trial, each attempt furnishes important evidence for or against its final adoption. The following outline of the case alluded to, is made from more extended notes recorded at the time:

In February, 1877, I was consulted by a young lady who, when a child, had fallen face foremost into an open fire, and the injury sustained had resulted in an unsightly cicatrix, which covered the right half of the forehead, drawing together the surrounding tissues and producing an ectropion of the upper lid of the most complete type. At least the outer third of the line of the lashes blended with the eyebrow, and the remaining portion was also displaced to such a degree as to expose almost the entire surface of the conjunctiva. The palpebral portion of this membrane had become swollen and thickened, but although it was quite impossible to close the eye, the ocular portion of the conjunctiva was not greatly injected, and the cornea remained entirely clear.

The patient also had convergent strabismus of the same eye, apparently of cicatricial origin, and the vision with + 30 cyl. 90° was only $\frac{20}{200}$. In this case it would have been a hopeless task to take a flap from the forehead or cheek and twist it into an eyelid, and the Italian method therefore appeared at first to offer the best prospects for success. As a matter of preliminary trial, the arm was simply fixed in the desired position by means of bandages, but after one restless night this arrangement was found to be sadly imperfect. An iron frame was then made, which, being fastened to the head, held the upraised arm firmly in place, but at the end of forty-eight hours the pain was so excessive that the patient begged to be released.

Meanwhile my attention had been directed to the operation of transplantation, which in the present instance seemed to be particularly appropriate.

Accordingly, on March 21, 1877, chloroform was administered to the patient, and after correcting the strabismus, I proceeded to bring down the lid, by making an incision parallel to the line of the lashes, and just above them, which extended from the outer to the inner angle of the eye. The edge thus liberated was drawn down into its original place, but the thickened conjunctiva persisted in rolling outward instead of inward, and threatened to become a serious obstacle to success. One end of a thread was therefore carried upward, transfixing the hypertrophied membrane in the vicinity of the cul-de-sac, and near the inner angle of the eye, while the other end, also armed with a needle, was passed through a corresponding point near its outer portion. The conjunctiva was thus bent upon itself and suspended as it were, over the thread, the extremities of which were attached to the forehead. Moreover, the strip containing the lashes was also fastened to the lower lid by three strong sutures, in order to avoid all unecessary motion of the parts. There was thus produced upon the outer surface of the upper lid an eliptical wound which measured rather more than 1¾ inch in its longitudinal diameter and over an inch tranversely. Its surface was even, free from fat, partially glazed, and bleeding very slightly.

Before commencing the operation a paper pattern of the desired flap had been made, and its outlines traced in ink upon the inner aspect of the right arm. It measured 3⅛ inches long by 1⅜ inches wide, being, of course, of eliptical shape, and this flap was carefully dissected up by an assistant, while the eye was being manipulated as above mentioned. Previous to the entire detachment of the piece, however, four fine sutures were introduced at the extremites of the principal diameters, for the sake of greater convenience in handling, and readiness in attaching it. The assistant was careful to free its surface from superfluous fat, and it was also placed upon the prepared spot without unnecessary delay.

It will be observed that the transplanted flap was very much larger than the space to be covered; but this allowance for contraction was none too great, and some slight difficulty was experienced in adjusting the edges, which were finally united by six or seven sutures in addition to the four already mentioned. The flap was then covered with a piece of gold-beater's skin which extended beyond the edges of the wound, and the region of the eye being thickly padded with charpie, a roller-bandage was applied. The outer dressings were not disturbed for forty-eight hours, when, on removing the charpie, the parts were found to be in the desired condition, and the skin could be seen of its normal color.

On the eleventh day, the stitches holding down the upper lid, together with the thread under the conjunctiva, were removed and the strabismus found to be entirely corrected. On the fourteenth day the inner half of the flap became discolored, and this appearance extended gradually over its entire surface, but involved only the epithelial layer, beneath which a new one had been produced. On the seventeenth day, however, the inner corner of the flap appeared to be more seriously threatened, and a small strip along the edge, measuring two lines in breadth and four in length, became dry and was detached. On the twenty-fifth of April—about one month after the operation—a photograph was taken of the patient, which illustrates the proportionately large size of the flap.

This continued to shrink, however, and two months later presented the appearance shown in the second photograph, being less than one and one-half inches long and three-eighths of an inch wide. In this, it will be noticed that the wound on the arm had healed after the usual manner of such granulating surfaces. The patient left Buffalo for her home in Iowa, July 1, 1877, and letters, since received, state that the good effects still continue as apparent as when the last photograph was taken. The movements of the lid are naturally much restricted, but otherwise the eye gives her no trouble, and looks infinitely better than at first.

From one such case it is evidently impossible to form any conclusions as to the requisites for success; but, comparing this single observation with the experience of the writers before mentioned, I should infer it were advisable—

As to the surface of the wound:

1. That it be clean, and free from adipose tissue.
2. That its base be even.

3. That there be little or no hemorrhage at the moment of transplantation.

Then, as to the flap:

1. That it be at least one-third larger than the wound.
2. That its inner surface be clean and free from fat.
3. That it be subjected to no undue violence.
4. That it be secured in its new position without delay.

Finally, as to adapting the parts to each other:

1. That the edges should fit evenly.
2. That they should be secured firmly.
3. That the parts should be kept warm and dry for at least twenty-four hours.

The advantages of this method are so evident as to require but few words of explanation:

1. A second scar is not produced in the attempt to rectify the deformity as by the Indian operation.
2. There is no pain caused by keeping the arm immovably fixed, as by the Italian operation.
3. The edges of the transplanted flap can be adjusted with the greatest nicety, which is not the case with either of these methods.
4. If the attempt should prove a complete failure, as a result of any accident, the deformity would remain virtually as at first.

In general, therefore, we have by it much to gain, and comparatively little to lose.

We are always in danger of judging too partially of any method or agent which has happened to serve us well. But, concerning this operation for the treatment of ectropion by transplantation of skin, when we see what satisfactory results have followed its employment in even a few cases, and when we remember, too, the great gains to be obtained, the subject would at least seem to be worthy of more frequent and more prominent mention in ophthalmic literature.

## Figures and Tables

**Figure f1:**
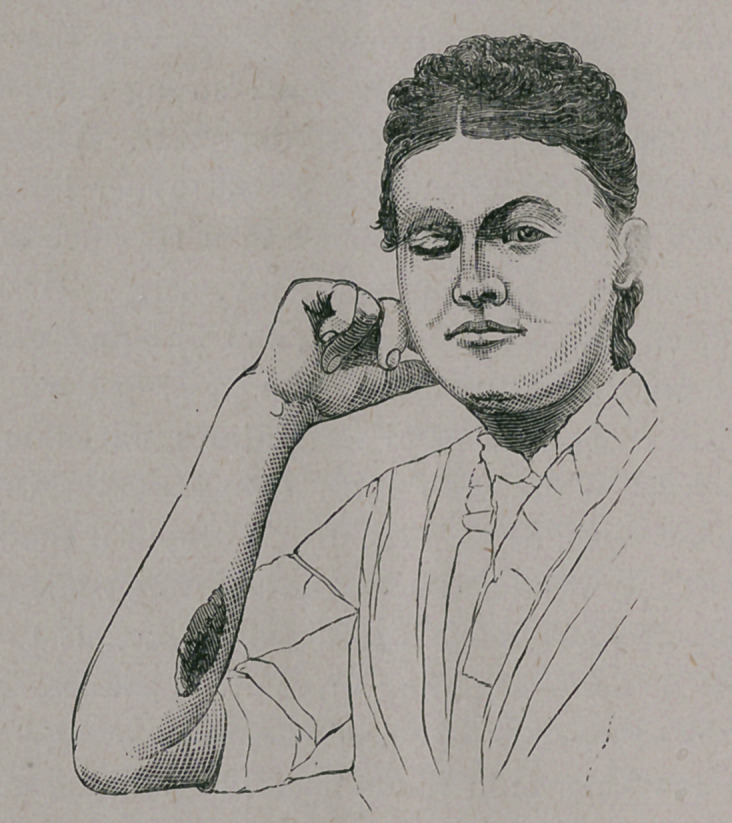


**Figure f2:**